# Epidemiologic aspects and spatial distribution of human visceral leishmaniasis: a cross-sectional study, Crateús, 2007-2023

**DOI:** 10.1590/S2237-96222025v34e20250155.en

**Published:** 2025-09-08

**Authors:** Ana Beatriz Dantas Pinto, Bárbara Vitória de Sousa Thomás, Lorena Maria Ribeiro Lima, José Cleves da Silva Maia, Antonio Ferreira Mendes de Sousa, Denise Barguil Nepomuceno

**Affiliations:** 1 Universidade Estadual do Ceará, Faculdade de Educação e Ciências Integradas de Crateús, Crateús, CE, Brazil; 2 Universidade Federal do Ceará, Sobral, CE, Brazil; 3 Fundação Universidade Federal do Vale do São Francisco, Petrolina, PE, Brazil; 4 Centro Universitário Unichristus, Fortaleza, CE, Brazil; 5 Universidade Federal do Piauí, Picos, PI, Brazil

**Keywords:** Neglected Diseases, Leishmaniasis, Visceral, Spatial Analysis, Cross-Sectional Studies, Epidemiologic Studies, Enfermedades desatendidas, Leishmaniasis visceral, Análisis espacial, Estudios transversales, Estudios epidemiológicos

## Abstract

**Objective:**

To investigate the epidemiological profile and spatial distribution of human visceral leishmaniasis cases in Crateús, Ceará, Brazil, from 2007 to 2023.

**Methods:**

This was a retrospective, descriptive cross-sectional study based on epidemiologic data from the Notifiable Health Conditions Information System regarding human visceral leishmaniasis in Crateús between 2007 and 2023. The variables assessed were sex, age group at the time of diagnosis, race/skin color, education level, HIV coinfection, case outcome, neighborhood of residence, area of residence, and number of deaths per year of notification. Relative frequency, incidence, and lethality rates were calculated; maps were created to reflect the spatial distribution of cases within the municipality.

**Results:**

Between 2007 and 2023, 112 cases of visceral leishmaniasis were reported in Crateús, predominantly among males (n=77), brown-skinned individuals (n=81), aged 20-39 years (n=27), with incomplete elementary education (n=31), and residing in urban areas of the municipality (n=88). The incidence rate decreased from 22.81 in 2007 to 16.02 in 2008, with a peak of 14.67 in 2018; by 2023, the incidence rate had dropped to 10.47. There were four cases of HIV coinfection and two deaths directly attributable to the disease.

**Conclusion:**

Cases of visceral leishmaniasis in Crateús were concentrated in the urban area of the municipality. The epidemiologic profile was consistent with national trends, with the most affected individuals being of mixed race/skin color, male, aged 20-39 years, and with incomplete elementary education.

## Resumen

**Objetivo:**

Investigar el perfil epidemiológico y la distribución espacial de los casos de leishmaniasis visceral humana en Crateús en el periodo 2007-2023.

**Métodos:**

Se trata de un estudio transversal, retrospectivo y descriptivo, basado en datos epidemiológicos procedentes del Sistema de Información de Enfermedades de Notificación Obligatoria relativos a la leishmaniasis visceral humana en Crateús entre 2007 y 2023. Se evaluaron el sexo, el rango de edad en el momento del diagnóstico, la raza/color de la piel, el nivel de escolaridad, la coinfección por VIH, la evolución del caso, el barrio de residencia, la zona de residencia y el número de muertes por año de notificación. Se calcularon la frecuencia relativa y las tasas de incidencia y letalidad; se elaboraron mapas para reflejar la distribución de los casos de la enfermedad en el municipio.

**Resultados:**

Entre 2007 y 2023, se produjeron 112 casos de leishmaniasis visceral en Crateús, predominantemente en individuos de sexo masculino (n=77), mestizos (n=81), en el rango de edad de 20 a 39 años (n=27), con educación primaria incompleta (n=31) y residentes en la zona urbana del municipio (n=88). Se observó una reducción de la tasa de incidencia del 22,81 en 2007 al 16,02 en 2008, destacando el aumento al 14,67 en 2018; en 2023, la tasa de incidencia fue del 10,47. Se observó coinfección con el VIH en cuatro casos. Se registraron dos fallecimientos como consecuencia directa de la enfermedad.

**Conclusión:**

Los casos de leishmaniasis visceral en Crateús se concentraron en la zona urbana del municipio. El perfil epidemiológico se mostró en línea con la tendencia nacional, siendo los individuos de raza parda, sexo masculino, edad comprendida entre 20 y 39 años y con estudios de enseñanza primaria incompletos los más afectados por la enfermedad.

**Ethical aspects**

This research respected ethical principles, having obtained the following approval data:

Research Ethics Committee: Universidade Estadual do Ceará

Opinion number: 6,629,460

Approval date: 30/1/2024

Certificate of Submission for Ethical Appraisal: 76460123.4.0000.5534

Informed Consent Form: Not applicable

## Introduction

Neglected tropical diseases are especially prevalent in tropical areas and represent a diverse group of 20 conditions that affect more than 1 billion people living in impoverished communities. These diseases have a strong socioeconomic component, as they predominantly affect the most vulnerable populations with limited access to health care around the world. Neglected tropical diseases have complex epidemiology, influenced by time and space; many of them are vector-borne and/or associated with animal reservoirs and complex biological cycles, which present a public health challenge. Among the 20 neglected tropical diseases is visceral leishmaniasis (1).

Most cases are reported in Brazil, India, and East Africa. Globally, between 50,000 and 90,000 cases of visceral leishmaniasis occur each year; however, only 25.0% to 45.0% of these cases are reported to the World Health Organization, which compromises the accuracy of global estimates (2). In Latin America, from 2001 to 2021, 69,665 new cases of visceral leishmaniasis were reported (2); in Brazil, between 2012 and 2021, 29,562 cases were registered, with an average of 3,284 cases per year (3). In Brazil, the primary species responsible for the etiology of human visceral leishmaniasis is *Leishmania infantum*, transmitted through the bite of infected female sand flies of the species *Lutzomyia longipalpis* (4).

Human visceral leishmaniasis is an insidious, debilitating disease with chronic progression (5), characterized by irregular fever episodes, weight loss, hepatomegaly and/or splenomegaly, and anemia. Children under 10 years of age and immunocompromised individuals are the groups most likely to develop severe forms of the disease (6). Also known as kala-azar, the disease is systemic, chronic, debilitating, and zoonotic. If left untreated, it can lead to death in more than 90.0% of cases (4). 

In Brazil, the epidemiological profile of the disease has been changing due to increasing and disordered urbanization, marked by irregular land use and precarious living conditions (7). In Fortaleza, cases of human visceral leishmaniasis have been concentrated in urban areas compared to other zones (8). Ceará, following the national trend, is endemic for human visceral leishmaniasis and, along with the states of Maranhão, Minas Gerais, Pará, and Tocantins, recorded the highest number of reported cases in 2021 (3). Between 2007 and 2022, 6,926 confirmed human cases were registered in Ceará, with an average of 433 cases per year, of which 86.7% (6,005 cases) were autochthonous. In 2019, 92 municipalities recorded incidence rates of up to 20 cases per 100,000 inhabitants, with an average of 6.1 cases. In 2021, 46 municipalities recorded up to 20 cases per 100,000 inhabitants (9).

Located in Ceará, Crateús is considered endemic for human visceral leishmaniasis. Although data are available at the level of regional health superintendencies, there is no recent data analysis specifically addressing the current epidemiological situation in this municipality. A specific approach focused on the municipality is essential to identify occurrence patterns, the most affected areas, and weaknesses in the surveillance of this endemic disease.

Given that human visceral leishmaniasis is a neglected tropical disease of significant public health relevance. With a strong socioeconomic component, regular investigation of the epidemiological profile of affected individuals is essential to guide control and surveillance measures. In line with Sustainable Development Goal 3 – good health and well-being – and the expansion of healthcare access in inland regions, this study aimed to investigate the epidemiological profile and spatial distribution of visceral leishmaniasis cases in Crateús from 2007 to 2023.

## Methods

### Design 

This was a cross-sectional, quantitative, retrospective, and descriptive epidemiological study based on secondary epidemiological data from confirmed cases of human visceral leishmaniasis recorded in the Notifiable Health Conditions Information System (SINAN) of the Brazilian Ministry of Health for the municipality of Crateús between 2007 and 2023.

### Setting

Crateús is a municipality in the state of Ceará, located in the Sertão dos Crateús microregion, approximately 350 km from the state capital, Fortaleza. According to the latest census conducted by the Brazilian Institute of Geography and Statistics in 2022, the municipality has a population of 76,390 inhabitants, an area of approximately 2,981.459 km^2^, and a municipal human development index (HDI) of 0.644 (10).

### Data sources and participants

The secondary data referring to confirmed cases of human visceral leishmaniasis were extracted from the Notifiable Health Conditions Information System through the Coordination of the Epidemiological Surveillance Unit of the Crateús Municipal Health Department. Given the nature of this study, there was no direct involvement of patients, and researchers had no access to or contact with any records or files containing personal information or the patients’ residential addresses, making it impossible to identify individuals. Data were collected between February and April 2024 and were previously organized and tabulated in a Microsoft Excel 2016 spreadsheet (Microsoft Corporation). 

### Variables

The variables assessed regarding person, space, and time included sex, age group at the time of diagnosis, race/skin color, education level, HIV coinfection, case outcome, neighborhood of residence, area of residence, and number of deaths per year of notification from 2007 to 2023. Data referring to cases registered outside the selected study period, incomplete or duplicate data, as well as data referring to patients diagnosed in Crateús but residing in other municipalities, were excluded.

### Statistical methods

GraphPad Prism software (version 9, Domatics) was used to calculate relative frequencies for the variables sex, age group, education level, race/skin color, area of residence, and HIV coinfection, and to calculate incidence and lethality rates for visceral leishmaniasis during the study period. The incidence rate was calculated by dividing the number of new cases of human visceral leishmaniasis by the population of Crateús per year and multiplying by 100,000 inhabitants. The lethality rate was calculated by dividing the number of deaths from visceral leishmaniasis in a given year by the number of existing cases that year, and then multiplying by 100. To create maps reflecting the spatial distribution of cases in the municipality, QGIS software version 2.14 (QGIS Development Team, Essen, Germany) was used. Demographic data were obtained from the Brazilian Institute of Geography and Statistics. 

## Results

**Figure 1:**
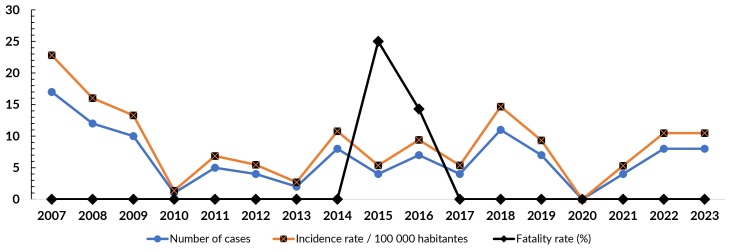
Number of cases, incidence rate, and case fatality rate of human visceral leishmaniasis. Crateús, 2007-2023 (n=112)

Between 2007 and 2023, 112 cases of human visceral leishmaniasis were reported in Crateús, with 2007 being the year with the highest number of cases (n=17). Throughout the historical series analyzed, a decrease in the number of cases was observed, starting in 2008 (n=12), and continuing until 2010, when only one case was confirmed in the municipality (Figure 1). A period of fluctuations in the number of reported cases followed, culminating in a new peak in 2018, when 11 cases were confirmed. Unlike the other years evaluated, there were no records in 2020. An apparent stabilization in the number of reported cases was observed, with eight records in 2022 and 2023. 

The incidence rate of visceral leishmaniasis decreased from 22.81 in 2007 to 16.02 in 2008, with a notable increase to 14.67 in 2018. In 2023, the last year analyzed, the incidence rate was 10.47. Regarding disease outcomes, 84.8% of the cases (n=95) progressed to a cure. Two deaths directly attributable to the disease were recorded during the study period, in 2015 (n=1) and 2016 (n=1), resulting in lethality rates of 25.0% and 14.3%, respectively (Figure 1).

Males were the most affected by the disease, accounting for 68.8% of cases (n=77), and brown-skinned individuals were the most impacted (72.3%, n=81) (Table 1). The highest number of cases was recorded among individuals aged 20–39 years (24.1%, n=27), although high case counts were also found in the 40–59 years (19.5%, n=22), 1–4 years (19.0%, n=21), and 5–9 years (9.4%, n=10) age groups. Most cases occurred among individuals with incomplete elementary education (27.7%, n=31); however, this information was unavailable for 57.1% of the cases (n=64). Regarding area of residence, 88 cases (78.6%) were confirmed in urban areas of the municipality.

**Table 1:** Distribution of human visceral leishmaniasis cases by sex, age group, education level, race/skin color, and area of residence. Crateús, 2007-2023 (n=112)

Variable	n (%)
Sex	
Female	35 ( 31.2)
Male	77 ( 68.8)
**Age group** (years)	
<1	7 ( 6.1)
1-4	21 ( 19)
5-9	10 ( 9.4)
10-14	4 ( 3.4)
15-19	3 ( 2.6)
20-39	27 ( 24.1)
40-59	22 ( 19.5)
60-69	8 ( 7.1)
70-79	5 ( 4.4)
>79	5 ( 4.4)
**Education level**	
Unknown/Blank	28 ( 25)
Illiterate	6 ( 5.3)
Incomplete elementary school	31 ( 27.7)
Complete elementary school	5 ( 4.5)
Incomplete high school	3 ( 2.7)
Complete high school	2 ( 1.8)
Incomplete higher education	1 ( 0.9)
Not applicable	36 ( 32.1)
**Race/skin color**	
White	18 ( 16)
Black	7 ( 6.3)
Asian	1 ( 0.9)
Brown	81 ( 72.3)
Unknown/Blank	5 ( 4.5)
**Residence Area**	
Urban	88 ( 78.6)
Rural	24 (21.4)

Clinical data revealed HIV coinfection in 3.6% (n=4) of cases, though this information was missing for 35.0% of patients (n=39). In Crateús, the epidemiological profile of human visceral leishmaniasis cases is characterized by a predominance among males, brown-skinned individuals, aged 20–39 years, with incomplete elementary education, and residents of urban areas.

Of the 112 cases of visceral leishmaniasis in Crateús from 2007 to 2023, 92.9% were reported as new cases, with no records of recurrence. Parasitological diagnosis was used to confirm 14.2% (n=16) of cases, while immunological diagnosis by indirect immunofluorescence was used in 30.3% (n=34) of cases. In 72.3% of cases (n=81), the initial treatment involved pentavalent antimonial. Liposomal amphotericin B was used sporadically.

**Figure 2:**
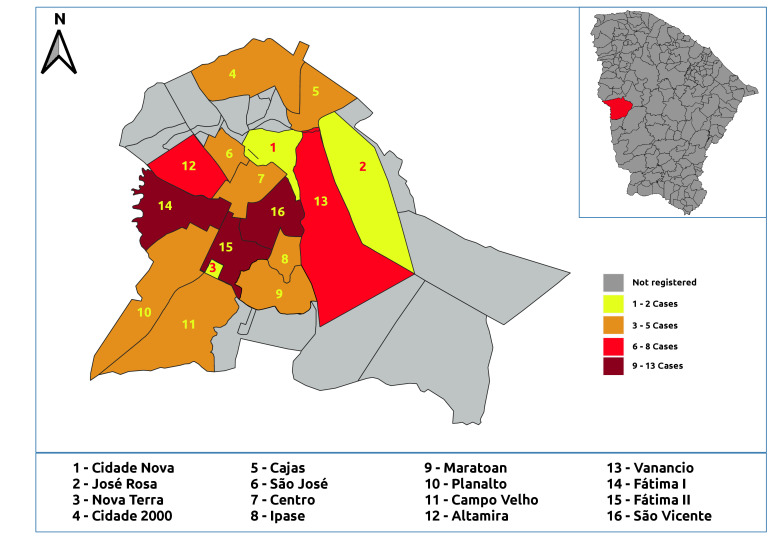
Distribution of human visceral leishmaniasis cases registered in urban areas. Crateús, 2007-2023 (n=88)

**Figure 3:**
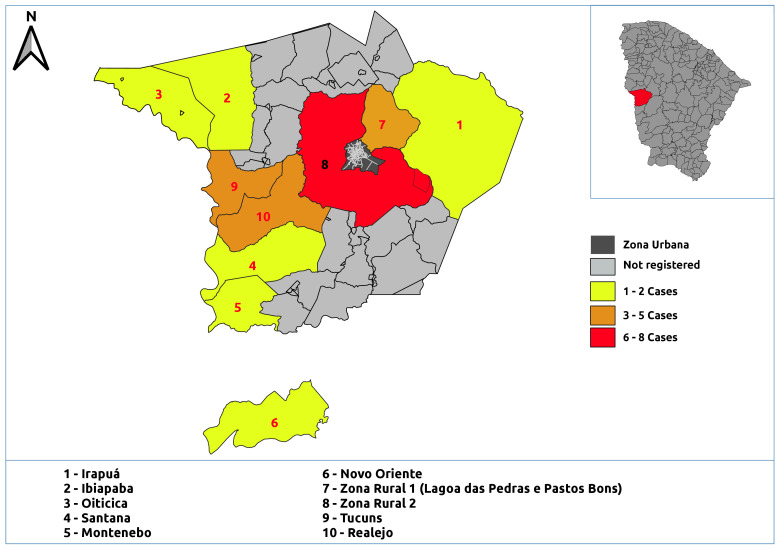
Distribution of human visceral leishmaniasis cases registered in rural areas. Crateús, 2007-2023 (n=24)

Cases were concentrated in the urban area, especially in the neighborhoods of Fátima II (11.6%, n=13), Fátima I (10.7%, n=12), São Vicente (9.8%, n=11), and Venâncios (7.1%, n=8) (Figures 2 and 3). 

## Discussion

In Crateús, the epidemiological profile of human visceral leishmaniasis cases is characterized by males, individuals of brown skin color, with incomplete elementary education, aged 20–39 years, and residing in urban areas. During the study period, two deaths were directly attributed to the disease, and four cases involved HIV coinfection. 

Given that the analyses were based on secondary data available in the Notifiable Health Conditions Information System, this study has some limitations. These include variables with high proportions of missing or blank data on notification forms, possible underreporting during the peak of the COVID-19 pandemic in 2020 and 2021, and occasional errors in form completion. These limitations may affect the situational diagnosis and distort associations between variables. Nevertheless, as visceral leishmaniasis is a notifiable disease, and reporting is required to access treatment medications, the impact of these factors on the analysis was minimal. The results obtained reflect the epidemiological scenario of visceral leishmaniasis over the 15 years analyzed.

In 2022, 1,684 cases of visceral leishmaniasis were confirmed in Brazil, with 731 cases in the Northeast region, and 189 of these cases occurred in Ceará (3). In northern Ceará, 1,689 cases of visceral leishmaniasis were confirmed between 2007 and 2023, with a notable concentration in Sobral and Crateús (11). In Crateús, the predominance of cases among males aged 20–59 years mirrors the national pattern of disease occurrence (3). This higher prevalence among males may be associated with a combination of biological (specifically hormonal) and occupational factors, since work activities often increase exposure to infected vectors (12,13).

Between 2011 and 2018, among the 204 municipalities in Ceará, Fortaleza had the highest number of reported visceral leishmaniasis cases, followed by Sobral, Juazeiro do Norte, Caucaia, Maracanaú, Itapipoca, Crato, Granja, Barbalha, and Várzea Alegre. Among the reported cases, the majority of affected individuals were male and aged 20–59 years, followed by children aged 0–9 years (14).

At the state level, the highest proportion of female cases occurred among children aged 1–9 years (11); in this analysis, this group was the second most affected. In 2017, Ceará recorded an incidence rate of 12.67 childhood cases per 100,000 inhabitants, particularly among boys under five living in urban areas (7). Children affected by visceral leishmaniasis are more susceptible to developing severe forms of the disease due to their immature immune system and/or the frequent presence of malnutrition in endemic areas. Without early diagnosis and timely treatment, these children account for a large proportion of fatalities (15,16). 

Most cases involved brown-skinned individuals with incomplete elementary education. Low educational levels may be associated with a lack of knowledge about preventive measures, contributing to higher incidence rates (13,17). Poor education leads to lower purchasing power and greater socioeconomic vulnerability, resulting in a strong relationship between visceral leishmaniasis and peripheral locations with low income and precarious infrastructure (18).

HIV coinfection was recorded in 3.6% of confirmed cases, a figure lower than national and state averages. From 2007 to 2023, 595 cases of Leishmania-HIV coinfection were recorded in Ceará, with notable peaks in 2021 and 2023, when coinfection occurred in 23.4% and 20.9% of cases, respectively (9). Considering the high number of records where this information was missing, HIV-Leishmania coinfection may be underreported in Crateús. 

Cases of the disease were concentrated in the urban area, particularly in the neighborhoods of Fátima I, Fátima II, São Vicente, and Venâncios. Initially considered a rural endemic or transitional zone disease, the urbanization of visceral leishmaniasis began in the 1980s, when urban outbreaks were recorded in various regions of Brazil (19). The spread and urbanization of visceral leishmaniasis have been observed across all regions of the country, a phenomenon linked to the ineffectiveness of current control and prevention measures in interrupting disease transmission (20).

In Sobral, a municipality in Ceará that is endemic for visceral leishmaniasis, 247 cases were reported between 2015 and 2018, with higher prevalence among males, brown-skinned individuals, with low education levels, and aged 0–4 years, followed by those aged 20–39 years (17,21). Along with Sobral and Fortaleza, the high number of cases detected in the Cariri region indicates that the disease is particularly prevalent in municipalities with high levels of urbanization and intense population mobility, which exacerbates anthropogenic environmental changes and affects the epidemiological profile of visceral leishmaniasis (7). A similar situation is observed in the state of Piauí, in municipalities such as Picos (12) and Altos (20).

The urbanization process in Crateús was driven by land concentration and declining rural job opportunities, which led to rural populations settling around the urban center (22). The city center experienced high population density, while the outskirts became occupied by precarious housing clusters marked by irregular paving, lack of sanitation, poor lighting, overgrown vegetation, and waste accumulation. The advance of urbanization, unplanned land use, environmental degradation, limited access to treatment, and lack of sanitation may contribute to the persistence of visceral leishmaniasis (20).

Climate change, particularly variations in temperature and rainfall, has also influenced the distribution of the disease by affecting the size and geographic range of sand fly populations. Additionally, droughts, famines, and floods impact migration flows, forcing people to relocate to areas with higher transmission rates (5).

Several factors have contributed to the intensification of visceral leishmaniasis in urban areas. Underreporting in rural areas due to a lack of resources and infrastructure, limited access to diagnostic services, changes in migration patterns, precarious socioeconomic conditions, and increased deforestation for housing, road, and factory construction have contributed to the growing number of cases in urban zones (13).

This study analyzed data on the occurrence of human visceral leishmaniasis in Crateús over 15 years. As Ceará is endemic for the disease, the findings reinforce the importance of periodically assessing the local situation. The results of this study will be shared with the municipal epidemiological surveillance coordination team, hence its relevance to the local context, where human and financial resources can be directed to priority areas, including entomological surveillance actions. The data can also support the development of training strategies that guide health professionals on how to properly complete notification forms, thereby helping to reduce epidemiological data gaps. 

Therefore, the epidemiological profile of the people most affected by the disease should be considered when developing public health education policies and prevention and control strategies. Spatial distribution maps can be used by health authorities to identify priority areas for intervention in the municipality and generate actions tailored to the specific characteristics of the region. 

## Data Availability

The dataset supporting the results of this study is not publicly available. They were obtained upon request, after approval by a research ethics committee, from the Epidemiological Surveillance Center of the Municipal Health Department of Crateús.
