# Supplementary material for: Epidemiologic aspects and spatial distribution of human visceral leishmaniasis: a cross-sectional study, Crateús, 2007-2023
Source: Epidemiol Serv Saude. 2025 Sep 8;34:e20250155. doi: 10.1590/S2237-96222025v34e20250155.en (PMC12424352; doi:10.1590/S2237-96222025v34e20250155.en)
Supplement: Translation

# Translation: Aspectos epidemiológicos e distribuição espacial da leishmaniose visceral humana: estudo transversal, Crateús, 2007-2023

**DOI:** 10.1590/S2237-96222025v34e20250155.pt

**Authors:** Ana Beatriz Dantas Pinto, Bárbara Vitória de Sousa Thomás, Lorena Maria Ribeiro Lima, José Cleves da Silva Maia, Antonio Ferreira Mendes de Sousa, Denise Barguil Nepomuceno

**Affiliations:** 1 Universidade Estadual do Ceará, Faculdade de Educação e Ciências Integradas de Crateús, Crateús, CE, Brasil; 2 Universidade Federal do Ceará, Sobral, CE, Brasil; 3 Fundação Universidade Federal do Vale do São Francisco, Petrolina, PE, Brasil; 4 Centro Universitário Unichristus, Fortaleza, CE, Brasil; 5 Universidade Federal do Piauí, Picos, PI, Brasil

**Keywords:** Doenças Negligenciadas, Leishmaniose Visceral, Análise Espacial, Estudos Transversais, Estudos Epidemiológicos

## Abstract

**Objetivo:**

Investigar o perfil epidemiológico e distribuição espacial dos casos de leishmaniose visceral humana em Crateús no período 2007-2023.

**Métodos:**

Tratou-se de estudo transversal, retrospectivo descritivo, baseado em dados epidemiológicos oriundos do Sistema de Informação de Agravos de Notificação referentes à leishmaniose visceral humana em Crateús de 2007 a 2023. Foram avaliados sexo, faixa etária do diagnóstico, raça/cor da pele, escolaridade, coinfecção com HIV, evolução do caso, bairro de residência, zona de residência e número de óbitos por ano de notificação. Foram calculadas a frequência relativa e as taxas de incidência e letalidade; mapas foram confeccionados para refletir a distribuição dos casos da doença no município.

**Resultados:**

Entre 2007 e 2023, ocorreram 112 casos de leishmaniose visceral em Crateús, predominantemente em indivíduos do sexo masculino (n=77), pardos (n=81), na faixa etária 20-39 anos (n=27), com ensino fundamental incompleto (n=31) e residentes na zona urbana do município (n=88). Houve redução da taxa de incidência de 22,81 em 2007 para 16,02 em 2008, destacando-se o aumento para 14,67 em 2018; em 2023, a taxa de incidência foi 10,47. Houve coinfecção com o HIV em quatro casos. Foram registrados dois óbitos em decorrência direta da doença.

**Conclusão.:**

Os casos de leishmaniose visceral em Crateús concentraram-se na zona urbana do município. O perfil epidemiológico mostrou-se alinhado à tendência nacional, sendo indivíduos pardos, sexo masculino, faixa etária 20-39 anos e com ensino fundamental incompleto os mais afetados pela doença.

**Aspectos éticos**

A presente pesquisa respeitou os princípios éticos, obtendo os seguintes dados de aprovação:

Comitê de ética em pesquisa: Universidade Estadual do Ceará

Número do parecer: 6.629.460

Data de aprovação: 30/1/2024

Certificado de apresentação de apreciação ética: 76460123.4.0000.5534

Registro do consentimento livre e esclarecido: Não se aplica.

## Introdução

As doenças tropicais negligenciadas são especialmente prevalentes em áreas tropicais e representam um grupo diverso de 20 agravos que afetam mais de 1 bilhão de habitantes de comunidades empobrecidas. Essas doenças têm forte caráter socioeconômico, pois atingem as comunidades mais vulneráveis e carentes de acesso à saúde ao redor do mundo. As doenças tropicais negligenciadas têm epidemiologia complexa, influenciada pelo tempo e espaço; muitas delas são de transmissão vetorial e/ou estão associadas a reservatórios animais e ciclos biológicos complexos, o que gera uma situação desafiadora na perspectiva de saúde pública. Entre as 20 doenças tropicais negligenciadas, está a leishmaniose visceral (1).

A maior parte dos casos é registrada no Brasil, na Índia e no leste africano. Entre 50 mil e 90 mil casos de leishmaniose visceral, ocorrem anualmente no mundo, porém apenas entre 25,0% e 45,0% são reportados à Organização Mundial da Saúde, o que compromete a acurácia das estimativas (2). Na América Latina, no período 2001-2021, houve 69.665 novos casos de leishmaniose visceral (2); no Brasil, entre 2012 e 2021, foram registrados 29.562 casos, com média de 3.284 casos ao ano (3). No Brasil, a principal espécie implicada na etiologia da leishmaniose visceral humana é *Leishmania infantum*, transmitida a partir da picada de fêmeas de insetos da espécie *Lutzomyia longipalpis* infectadas pelo protozoário (4).

A leishmaniose visceral humana é uma doença insidiosa, debilitante e de evolução crônica (5), caracterizando-se por acessos febris irregulares, perda de peso, hepato e/ou esplenomegalia e anemia. Crianças menores de 10 anos e indivíduos imunocomprometidos são os grupos mais propensos a evoluírem para as formas graves da doença (6). Também chamada de calazar, a doença é sistêmica, de evolução crônica e debilitante, e de caráter zoonótico. Quando não tratada, pode evoluir para óbito em mais de 90,0% dos casos (4). 

No Brasil, o perfil epidemiológico da doença vem se modificando devido à urbanização crescente e tumultuosa, acentuada pela ocupação desordenada do solo e pelas precárias condições de vida (7). Em Fortaleza, os casos de leishmaniose visceral humana têm se concentrado na zona urbana em relação às demais zonas (8). O Ceará, seguindo a tendência nacional, é endêmico para a leishmaniose visceral humana e, junto aos estados do Maranhão, Minas Gerais, Pará e Tocantins, registrou os maiores números de casos reportados em 2021 (3). Entre 2007 e 2022, foram registrados 6.926 casos humanos confirmados, com média de 433 casos ao ano, dos quais 86,7% (6.005 casos) foram autóctones. Em 2019, 92 municípios registraram coeficientes de incidência de até 20 casos por 100 mil habitantes, com média de 6,1 casos. Em 2021, 46 municípios registraram até 20 casos por 100 mil habitantes (9).

Localizado no Ceará, Crateús é considerado endêmico para a leishmaniose visceral humana. Apesar de existirem informações quanto à ocorrência da doença em termos de superintendência regional de saúde, não há análise de dados recentes direcionada à atual situação epidemiológica nesse município. Uma abordagem específica para o município é essencial para identificar padrões de ocorrência, as áreas mais expostas e as fragilidades na vigilância dessa endemia.

Sendo a leishmaniose visceral humana uma doença tropical negligenciada de extrema relevância em saúde pública e com forte caráter socioeconômico, a investigação periódica do perfil epidemiológico de indivíduos acometidos é fundamental para direcionar medidas de controle e vigilância. Na perspectiva do Objetivo de Desenvolvimento Sustentável 3 – saúde e bem-estar – e da interiorização do acesso à saúde, este estudo teve como objetivo investigar o perfil epidemiológico e a distribuição espacial dos casos de leishmaniose visceral em Crateús no período 2007-2023.

## Métodos

### Delineamento 

Tratou-se de estudo epidemiológico transversal, quantitativo, retrospectivo e descritivo, baseado no uso de dados epidemiológicos secundários referentes aos casos de leishmaniose visceral humana confirmados no Sistema de Informação de Agravos de Notificação do Ministério da Saúde para Crateús no período 2007-2023.

### Contexto

Crateús é município do Ceará, localizado na microrregião do Sertão dos Crateús e a 350 km da capital, Fortaleza. Segundo o último censo do Instituto Brasileiro de Geografia e Estatística, realizado em 2022, o município possui população de 76.390 habitantes, área aproximada de 2.981,459 km^2^ e índice de desenvolvimento humano municipal de 0,644 (10).

### Fonte de dados e participantes

Os dados secundários referentes aos casos confirmados de leishmaniose visceral humana foram extraídos do Sistema de Informação de Agravos de Notificação por intermédio da Coordenação do Núcleo de Vigilância Epidemiológica da Secretaria Municipal de Saúde de Crateús. Considerando a natureza deste estudo, não houve envolvimento direto dos pacientes, e os pesquisadores não tiveram acesso/contato com qualquer registro/ficha que pudesse remeter à informação nominal ou à localização de residência dos pacientes, não havendo a possibilidade de identificação de tais pacientes. Os dados foram coletados de fevereiro a abril de 2024, previamente organizados e tabulados em planilha do software Microsoft Excel 2016 (Microsoft Corporation). 

### Variáveis

As variáveis relacionadas a pessoa, espaço e tempo avaliadas foram sexo, faixa etária do diagnóstico, raça/cor da pele, escolaridade, coinfecção com HIV, evolução do caso, bairro de residência, zona de residência e número de óbitos por ano de notificação entre 2007 e 2023. Dados referentes a casos registrados fora do período selecionado para estudo, incompletos ou duplicados, bem como dados referentes a pacientes diagnosticados em Crateús, porém residentes em outros municípios, foram excluídos.

### Métodos estatísticos

O software GraphPad Prism (versão 9, Domatics) foi utilizado para cálculo da frequência relativa – para as variáveis sexo, faixa etária, escolaridade, raça/cor da pele, zona de residência e coinfecção com vírus HIV – e das taxas de incidência e letalidade referentes aos casos de leishmaniose visceral para o período selecionado. A taxa de incidência foi calculada dividindo-se o número de novos casos de leishmaniose visceral humana pela população de Crateús por ano para 100 mil habitantes. A taxa de letalidade foi calculada dividindo-se o número de óbitos por leishmaniose visceral em determinado ano pelo número de doentes existentes nesse ano multiplicado por 100. Para a confecção de mapas que refletissem a distribuição dos casos da doença no município, foi utilizado o software QGis 2.14 (QGis Development Team, Essen-Germany). Os dados demográficos foram obtidos mediante consulta ao Instituto Brasileiro de Geografia e Estatística. 

## Resultados

Entre 2007 e 2023, ocorreram 112 casos de leishmaniose visceral humana em Crateús, sendo 2007 o ano com o maior número de casos (n=17). Ao longo da série histórica analisada, notou-se a redução no número de casos a partir de 2008 (n=12) até 2010, quando apenas um caso foi confirmado no município (Figura 1). Iniciou-se, então, um período de oscilações nos registros até que um novo pico ocorreu em 2018, quando 11 casos foram confirmados. Destoando dos demais anos avaliados, em 2020 não houve registros. Observou-se aparente estabilização no número de casos reportados, com oito registros em 2022 e em 2023. 

**Figura 1:** Número de casos, taxa de incidência e taxa de letalidade da leishmaniose visceral humana. Crateús, 2007-2023 (n=112)

Houve redução da taxa de incidência da leishmaniose visceral de 22,81 em 2007 para 16,02 em 2008, destacando-se o aumento para 14,67 em 2018. Em 2023, último ano avaliado, a taxa de incidência foi 10,47. Referente à evolução da doença, houve cura em 84,8% dos casos (n=95). No período avaliado, foram registrados dois óbitos em decorrência direta da doença, em 2015 (n=1) e 2016 (n=1), levando à taxa de letalidade de 25,0% e 14,3% (Figura 1).

O sexo masculino foi o mais acometido pela doença, respondendo por 68,8% dos casos (n=77), e indivíduos de raça/cor da pele parda foram os mais afetados (72,3%, n=81) (Tabela 1). O maior número de casos foi registrado em indivíduos de 20-39 anos (24,1%, n=27), mas também houve elevado número de casos nas faixas etárias 40-59 anos (19,5%, n=22), 1-4 anos (19,0%, n=21) e 5-9 anos (9,4%, n=10). Os casos concentraram-se em indivíduos com ensino fundamental incompleto (27,7%, n=31); contudo, essa informação estava indisponível para 57,1% dos indivíduos (n=64). Em relação à zona de residência, 88 casos (78,6%) foram registrados e confirmados na zona urbana do município.

**Tabela 1:** Distribuição dos casos de leishmaniose visceral humana, segundo sexo, faixa etária, escolaridade, raça/cor da pele e zona de residência. Crateús, 2007-2023 (n=112)

Variável	n (%)
Sexo	
Feminino	35 (31,2)
Masculino	77 (68,8)
**Faixa etária** (anos)	
<1	7 (6,1)
1-4	21 (19)
5-9	10 (9,4)
10-14	4 (3,4)
15-19	3 (2,6)
20-39	27 (24,1)
40-59	22 (19,5)
60-69	8 (7,1)
70-79	5 (4,4)
>79	5 (4,4)
**Escolaridade**	
Ignorado/em branco	28 (25)
Analfabeto	6 (5,3)
Ensino fundamental incompleto	31 (27,7)
Ensino fundamental completo	5 (4,5)
Ensino médio incompleto	3 (2,7)
Ensino médio completo	2 (1,8)
Ensino superior incompleto	1 (0,9)
Não se aplica	36 (32,1)
**Raça/cor da pele**	
Branca	18 (16)
Preta	7 (6,3)
Amarelo	1 (0,9)
Parda	81 (72,3)
Ignorado/em branco	5 (4,5)
**Zona de residência**	
Urbana	88 (78,6)
Rural	24 (21,4)

Os dados clínicos mostraram o registro de coinfecção com o vírus HIV em 3,6% (n=4) dos casos, sendo importante ressaltar que essa informação foi ignorada para 35,0% dos pacientes (n=39). É possível afirmar que, em Crateús, o perfil epidemiológico dos casos de leishmaniose visceral humana é caracterizado pelo acometimento predominante de indivíduos do sexo masculino, pardos, na faixa etária 20-39 anos, com 5ª a 8ª série do ensino fundamental incompleta e residentes na zona urbana do município.

Dos 112 casos de leishmaniose visceral em Crateús no período 2007-2023, 92,9% foram reportados como casos novos, não havendo registros de recorrência. O diagnóstico parasitológico foi usado para confirmação em 14,2% (n=16) dos casos, e o diagnóstico imunológico, por imunofluorescência indireta em 30,3% (n=34) dos casos. Em 72,3% dos casos (n=81), a droga utilizada como tratamento inicial foi o antimonial pentavalente. Esporadicamente, recorreu-se ao uso de anfotericina B lipossomal.

Os casos concentraram-se na zona urbana do município, com destaque para os bairros Fátima II (11,6%, n=13), Fátima I (10,7%, n=12), São Vicente (9,8%, n=11) e Venâncios (7,1%, n=8) (Figuras 2 e 3). 

**Figura 2:** Distribuição dos casos de leishmaniose visceral humana registrados na zona urbana. Crateús, 2007-2023 (n=88)

**Figura 3:** Distribuição dos casos de leishmaniose visceral humana registrados na zona rural. Crateús, 2007-2023 (n=24)

## Discussão

Em Crateús, o perfil epidemiológico dos casos de leishmaniose visceral humana é representado por indivíduos do sexo masculino, pardos, com ensino fundamental incompleto, faixa etária 20-39 anos e residentes na zona urbana do município. No período avaliado, foram registrados dois óbitos em decorrência direta da doença e quatro casos de coinfecção leishmaniose visceral-HIV. 

Considerando que as análises se basearam em dados secundários disponíveis no Sistema de Informação de Agravos de Notificação, este estudo apresentou algumas limitações. Destacaram-se as variáveis que apresentaram considerável percentual de dados ignorados ou deixados em branco quando do preenchimento das fichas de notificação, a possível subnotificação de casos no ápice da pandemia de covid-19 em 2020 e 2021 e as eventuais falhas de preenchimento das fichas. Tais limitações podem afetar o diagnóstico situacional da doença e distorcer associações entre variáveis. Entretanto, sendo a leishmaniose visceral uma doença de notificação compulsória e que precisa ser notificada para o repasse dos fármacos necessários ao tratamento, a interferência desses fatores nesta análise foi mínima, e os resultados obtidos refletiram o panorama epidemiológico da leishmaniose visceral durante o espaço temporal considerado.

Em 2022, foram confirmados no Brasil 1.684 casos de leishmaniose visceral, dos quais 731 casos foram na região Nordeste do país e, destes, 189 foram no Ceará (3). Na região norte do Ceará, foram confirmados 1.689 casos de leishmaniose visceral entre 2007 e 2023, com destaque para Sobral e Crateús (11). Em Crateús, o maior acometimento de indivíduos do sexo masculino e na faixa etária 20-59 anos replica o padrão nacional de ocorrência da doença (3). O maior acometimento do sexo masculino estaria relacionado à conjunção de fatores biológicos – especificamente fatores hormonais – e ocupacionais – pois, devido às suas atividades laborais, estariam mais expostos ao vetor infectado (12,13).

No período 2011-2018, entre os 204 municípios de residência do Ceará, Fortaleza concentrou o maior número de casos de leishmaniose visceral notificados, seguido por Sobral, Juazeiro do Norte, Caucaia, Maracanaú, Itapipoca, Crato, Granja, Barbalha e Várzea Alegre. Entre os casos notificados, houve maior acometimento de indivíduos do sexo masculino e na faixa etária 20-59 anos, seguido por crianças na faixa etária 0-9 anos (14).

Em nível estadual, a maior proporção de casos no sexo feminino ocorreu na faixa etária de 1-9 anos (11); nesta análise, essa faixa etária representou o segundo grupo etário mais afetado pela doença. Em 2017, foi registrada taxa de 12,67 casos infantis por 100 mil habitantes no Ceará, especialmente em menores de 5 anos do sexo masculino e residentes em áreas urbanas (7). Crianças afetadas pela leishmaniose visceral são mais susceptíveis à evolução para formas graves, o que é atribuído ao sistema imunológico imaturo e/ou à condição de desnutrição frequentemente encontrada em áreas endêmicas para a doença. Na ausência de diagnóstico precoce e tratamento oportuno, isso representa grande parte dos indivíduos que vão a óbito (15,16). 

Indivíduos da raça/cor da pele parda e com ensino fundamental incompleto responderam pela maioria dos casos. O baixo nível educacional estaria associado ao desconhecimento de medidas preventivas contra a leishmaniose visceral, o que favorece a maior incidência de casos (13,17). A baixa escolaridade levaria ao menor poder aquisitivo e à maior vulnerabilidade socioeconômica, de forma que haveria forte relação entre a leishmaniose visceral e as localizações periféricas, de baixa renda e infraestrutura precária (18).

Houve registro da coinfecção com HIV em 3,6% dos casos confirmados, abaixo do percentual registrado em nível nacional e estadual. No período 2007-2023, houve 595 casos de coinfecção Leishmania-HIV no Ceará, com destaque para os anos de 2021 e 2023, quando foi registrada a coinfecção em 23,4% e 20,9% dos casos (9). Considerando a elevada quantidade de casos em que essa informação foi ignorada, é possível que a coinfecção Leishmania-HIV esteja subnotificada em Crateús. 

Os casos da doença se concentraram na zona urbana do município, especialmente nos bairros Fátima I, Fátima II, São Vicente e Venâncios. De início considerada endemia rural ou de áreas de transição entre áreas de florestas/matas e zona urbana, o processo de urbanização da leishmaniose visceral teve início na década de 1980, a partir do que epidemias urbanas passaram a ser registradas em diversas regiões do país (19). O aumento da distribuição e da urbanização da leishmaniose visceral é observado em todas as regiões do Brasil, fato associado à ineficácia das atuais medidas de controle e prevenção em interromper o ciclo de transmissão da doença (20).

Em Sobral, município cearense endêmico para a leishmaniose visceral, foram notificados 247 casos da doença entre 2015 e 2018, com maior prevalência em indivíduos do sexo masculino, raça/cor da pele parda, com baixa escolaridade e na faixa etária 0-4 anos, seguida pela faixa etária 20-39 anos (17,21). Junto a Sobral e Fortaleza, a elevada detecção de casos na região do Cariri mostra que a doença está presente principalmente em municípios com elevado índice de urbanização e intenso fluxo de pessoas, o que favorece a ação antrópica no ambiente e o impacto no perfil epidemiológico da leishmaniose visceral (7). Situação similar é encontrada no Piauí, como nos municípios de Picos (12) e Altos (20).

O processo de urbanização de Crateús foi impulsionado pela concentração fundiária e pela crise do esgotamento de oportunidades de trabalho no campo, de modo que essa população passou a orbitar em torno do centro urbano (22). O centro da cidade viveu grande adensamento e as periferias passaram a concentrar casebres e pequenos aglomerados marcados por calçamento irregular, sistema de saneamento inexistente, iluminação precária, mato e acúmulo de lixo. O avanço do processo de urbanização, a ocupação desordenada e a exploração predatória do ambiente, além do acesso limitado ao tratamento e da falta de sanitização, podem contribuir para a prevalência da leishmaniose visceral (20).

Mudanças climáticas – especialmente as alterações de temperatura e precipitação de chuvas – também têm influenciado a distribuição da doença, o que afeta o tamanho e a distribuição geográfica das populações de flebotomíneos. Além disso, seca, fome e inundações impactam os fluxos migratórios, levando pessoas a áreas onde as taxas de transmissão são elevadas (5).

Diversos fatores têm colaborado para a intensificação do processo de urbanização da leishmaniose visceral. A subnotificação em áreas rurais em decorrência da falta de recursos e infraestrutura, a indisponibilidade do diagnóstico, as alterações nos fluxos migratórios, as condições socioeconômicas precárias e o aumento do desflorestamento para construção de casas, estradas e fábricas contribuem para o aumento de casos da doença em zonas urbanas (13).

Neste estudo, foram analisados os dados referentes à ocorrência da leishmaniose visceral humana em Crateús durante 15 anos. Sendo o Ceará endêmico para a doença, este estudo reforçou a necessidade da avaliação periódica da situação da leishmaniose visceral em Crateús. Os resultados deste estudo serão compartilhados com a coordenação de vigilância epidemiológica do município e nesse ponto encontra-se a sua relevância para o contexto local, quando recursos humanos e financeiros podem ser direcionados a áreas prioritárias, incluindo ações de vigilância entomológica. Os dados também podem ser utilizados para direcionar estratégias de capacitação e treinamento que orientem os profissionais da saúde quanto ao correto preenchimento das fichas de notificação, o que contribuirá para minimizar lacunas epidemiológicas. 

Portanto, o perfil epidemiológico das pessoas majoritariamente afetadas pela doença deve ser considerado na elaboração de políticas públicas de educação popular em saúde e de estratégias de prevenção e controle. Os mapas de distribuição espacial podem ser utilizados pelas autoridades de saúde para identificar as áreas prioritárias de intervenção no município e gerar ações direcionadas às particularidades da região. 

## Data Availability

O conjunto de dados que dá suporte aos resultados deste estudo não está disponível publicamente, tendo sido obtido mediante solicitação, após a devida aprovação em comitê de ética em pesquisa, ao Núcleo de Vigilância Epidemiológica da Secretaria Municipal de Saúde de Crateús.
